# Folic Acid Has a Protective Effect on Retinal Vascular Endothelial Cells against High Glucose

**DOI:** 10.3390/molecules23092326

**Published:** 2018-09-12

**Authors:** Zhenglin Wang, Wei Xing, Yongli Song, Hongli Li, Yonggang Liu, Yong Wang, Chun Li, Yun Wang, Yan Wu, Jing Han

**Affiliations:** 1 College of Traditional Chinese Medicine, Beijing University of Chinese Medicine, Beijing 100029, China; 20160931147@bucm.edu.cn (Z.W.); 18810252377@163.com (W.X.); 20170931140@bucm.edu.cn (Y.S.); mmmmmmlhl@163.com (H.L.); 2 School of Traditional Chinese Material Medica, Beijing University of Chinese Medicine, Beijing 100029, China; liuyg0228@163.com; 3 College of Life Science, Beijing University of Chinese Medicine, Beijing 100029, China; doctor_wangyong@sina.com; 4 Modern Research Center of Traditional Chinese Medicine, Beijing University of Chinese Medicine, Beijing 100029, China; lichun19850204@163.com; 5 Institute of Chinese Medicine, Beijing University of Chinese Medicine, Beijing 100029, China

**Keywords:** diabetic retinopathy, folic acid, retinal vascular endothelial cells, YAP1, TEAD1

## Abstract

Diabetic retinopathy (DR) is a severe complication of diabetes, which seriously affects the life quality of patients. Because of the damage caused by DR, there is an urgent need to develop effective drugs. Folic acid, a water-soluble vitamin, is one of the vitamin B complexes. Folic acid is widely found in the meat and vegetables. In the clinic, low folic acid levels in the body may have a certain correlation with DR. However, there is no relevant basic research proving a relationship between folic acid levels and DR. The purpose of this study was therefore to investigate whether folic acid has a protective effect on the retinal vascular endothelial cells against high glucose levels. Moreover, the molecular mechanism of action of folic acid was further explored. The results showed that folic acid significantly suppressed the cell viability, tube length, migrated cells and the percentage of BrdU^+^ cells compared with the high glucose group. Moreover, folic acid decreased the mRNA expression of TEAD1 and the protein expression of TEAD1 and YAP1. These findings indicate that folic acid can protect retinal vascular endothelial cells from high glucose-induced injury by regulating the proteins in the Hippo signaling pathway.

## 1. Introduction

Diabetic retinopathy (DR) which is characterized by the dysfunction of retinal vessels, capillary loss and endothelial cell damage is one of the most important complications of diabetes mellitus [1]. It causes irreversible vision loss in working populations [2,3]. According to the reports, approximately 16 million Americans will suffer from DR by 2050 [4]. Obviously, DR has already become an important public health problem, therefore, developing effective drugs to alleviate DR is considered a most important step.

Folic acid is a form of water-soluble vitamin B. Folic acid is composed of pteridine, *p*-aminobenzoic acid and L-glutamic acid. Folic acid exists in fresh fruits, vegetables and meat [5]. Folic acid is mainly present in the form of polyglutamic acid in food and organisms, while in drugs, supplements and fortified food, it is mainly present in the form of monoglutamic acid. Folic acid can’t be synthesized in the body but can be obtained from food. High intake of folic acid can reduce not only the cytotoxicity of natural killer cells, but also the body’s response to drugs against cancer, arthritis and so on [6].

Many clinical studies have shown that folic acid is closely related to DR. For example, Malaguamera et al. found the patients with proliferative diabetic retinopathy (PDR) or non-proliferative diabetic retinopathy (NPDR) had lower folic acid plasma levels compared with a group of diabetics without retinopathy (DWR) [7]. Furthermore, the deficiency of vitamins B_9_, B_12_ and B_6_ might increase the level of homocysteine [8]. Elevated homocysteine levels were associated with an increased risk of DR [9]. Besides, the combination of vitamins B_6_, B_9_ and B_12_ had some beneficial effects on NPDR [10]. However, there is no relevant basic research on the relationship between folic acid and DR.

In this study, we investigated whether folic acid had a protective effect on retinal vascular endothelial cells against the high glucose levels. In in vitro experiments, folic acid could decrease the cellular activity of the retinal vascular endothelial cells, inhibit migration and proliferation, and reduce the total length in the tube formation test.

We further explored the molecular mechanism of action of folic acid. The Hippo and Notch signaling pathways are closely related to angiogenesis, which is the primary pathological change of DR. If the Hippo signaling pathway is not activated, it will promote cell proliferation, cell migration and invasion. When the Notch signaling pathway is activated, it will induce a series of molecular responses that regulate cell proliferation, differentiation, migration and apoptosis. Therefore, we selected the Hippo and Notch signaling pathways for research. The results displayed that folic acid reduced the expression level of YAP1 and TEAD1 protein. In conclusion, our research manifests that folic acid probably has a protective effect on retinal vascular endothelial cells against high glucose levels by regulating the proteins in the Hippo signaling pathway.

## 2. Materials and Methods

### 2.1. Compound

Folic acid was purchased from Yuanye Bio-Company (B21487, Yuanye, China).

### 2.2. Cell Culture

The retinal vascular endothelial cell (RVEC) line was purchased from the Shanghai Institutes for Biological Sciences (Shanghai, China) and grown in the complete culture medium, which was composed of RMPI 1640 (Thermo Fisher Scientific, Grand Island, NY, USA), 10% fetal bovine serum (Corning, Manassas, VA, USA), 100 U/mL of penicillin and 100 μg/mL of streptomycin (Corning). According to the manufacturer’s instruction, the cells were maintained in the humidified incubator under 37 °C and 5% CO_2_. The medium was changed every 2–3 days.

### 2.3. Cytotoxicity Assay

The CCK-8 assay was performed to assess the cytotoxicity of folic acid. RVECs were seeded into 96-well plates at a density of 4000 per/well and allowed to grow for 24 h. After starvation for 24 h, the cells were exposed to different concentrations of folic acid. The RVECs grouped as controls were cultured in a medium with a glucose concentration at 5.5 mM. After an additional 72 h, the medium was replaced with fresh serum-free medium and 10 μL of CCK-8 solution (CCK-8; Dojindo Laboratories, Kumamoto, Japan) was added to each well. Then the plates were cultured at 37 °C for 2 h. The optical density (OD) was measured at 450 nm by using multi-detection plate reader (Spectramax M4, Molecular Devices, CA, USA).

### 2.4. Cell Viability Assay

The procedures were roughly the same as the cytotoxicity assay. However, the cell treating medium was different. In the cell viability assay, the medium was 5.5 mM of glucose, 25 mM of glucose or 25 mM of glucose plus folic acid.

### 2.5. Migration Assay

The cell migration was assayed by Transwell (Corning) with a pore size of 8.0 μm. Briefly, the cells (4 × 10^5^ cells/mL) were seeded on the upper chamber of inserts and 600 μL of different medium was added into the lower chamber. All migration assays were conducted at 37 °C for 16 h. After being fixed with 4% paraformaldehyde (Solarbio, Beijing, China), the RVECs on the upper side of the insert were scraped off gently with a cotton swab and washed with PBS. At the end of the assay, the cells were stained with 4′,6-diamidino-2-phenylindole (DAPI, Solarbio) for 10 min. The cells that migrated to the bottom side of the insert were photographed.

### 2.6. Tube Formation Assay

Tube formation assay estimates the ability of the cells to form capillary-like structures, which is convenient and quantifiable. The 96-well plates were coated with chilled Matrigel (Corning). And then the plates were incubated at 37 °C for 30 min. The RVECs (2 × 10^5^ cells/mL) treated with different culture media were seeded on the Matrigel for 16 h. The capillary-like structures in Matrigel were photographed.

### 2.7. BrdU Assay

The cells (5 × 10^4^ cells/mL) were seeded into the 24-well plates, which were placed in a humidified atmosphere of 5% CO_2_ for 24 h. After starvation, the RVECs were cultured in different media for another 72 h. BrdU was added to the culture medium 2 h before the cells were fixed with 4% paraformaldehyde. After 30 min, the cells were incubated with TritonX-100 (Amresco, Solon, OH, USA) and 2 N of HCl. The cells were washed with PBS after neutralization with Na_2_B_4_O_7_. The pre-treated cells were coated with Anti-BrdU antibody (1:100, Ab6326-ABC; Abcam, Cambridge, UK) at 4 °C overnight. After incubation with anti-rat-FITC secondary antibody (1:200, ZF-0315; Zsbio Commerce Store, Beijing, China) for 2 h at room temperature (RT), the cells were stained with DAPI. The images of positive cells were photographed with a laser confocal microscope.

### 2.8. Molecular Docking

To clarify the molecular mechanism of folic acid promptly, we used molecular docking to screen the proteins [11]. Molecular docking is strictly followed with Fisher’s “lock and key” principle [12]. When the proteins are matched in conformation and energy and form a stable ligand-receptor complexes, it probably proves that these small molecules may be agonists or inhibitors of the protein targets [12]. The crystal structures of the proteins in the Hippo and Notch signaling pathways, including MST1, YAP1, Lats, TAZ, TEAD, DLL1, Notch1 and Notch2 were downloaded from the RCSB Protein Data Bank (PDB).

According to the “lock and key” principle, the LeDock software (version 1.0, University of Zurich, Zurich, Switzerland) was used for molecular docking. In the docking process, the chemical constituent was employed as the ligands and the target proteins were employed as the receptors.

In a comprehensive docking program study, LeDock exhibited a higher accuracy and a faster speed than other sorts of software [13]. In LeDock, taking into account the protonation state of histidine, the hydrogen atoms were automatically added to the protein. Meanwhile, it could also eliminate all the small ligands, crystal water, cofactors and ions when the PH values equaled to 7. To avoid forming the redundant conformation, the RMSD value was defined as 1.0. The parameters of 20 conformations were set to the default values [14]. Then the proteins with higher docking scores were selected for verification.

### 2.9. Western Blot Analysis

To confirm the expression of the proteins in Hippo and Notch signaling, the proteins isolated from the RVECs were subjected to WB analysis. The cells were stimulated with 5.5 mM of glucose, 25 mM of glucose or 25 mM of glucose plus folic acid for 3 days, respectively. Afterwards, the total protein was extracted using a lysis buffer containing sodium dodecyl sulfate (SDS; Biodee Biotechnology Company, Beijing, China), dichlorodiphenyltrichloroethane (DDT; Biodee Biotechnology Co.), Tris-HCl (Applygen Technologies Inc., Beijing, China) and glycerol (Applygen Technologies Inc.). The cell lysates were loaded into the SDS-PAGE, and separated by electrophoresis. Then the proteins were transferred onto the PVDF membranes. After being blocked with 5% nonfat milk for 2 h at RT, the membranes were incubated overnight at 4 °C with anti-YAP1 (1:1000, ab39361; Abcam), anti-P-YAP1 (1:10,000, ab76252; Abcam), anti-TEAD1 (1:2000, ab133533; Abcam), anti-DLL1 (1:300, ab85346; Abcam), anti-Notch2 (1:500, ab8926; Abcam) or anti-β-actin (1:2000, sc-47778; Santa Cruz, Dallas, TX, USA). The following day, the membranes were washed with TBST and incubated with the secondary antibodies, which were anti-rabbit IgG (1:2000 in the Secondary Antibody Dilution Buffer; TOYOBO Co., Osaka, Japan), anti-goat IgG (1:2000 in the Secondary Antibody Dilution Buffer; TOYOBO Co.) or anti-mouse IgG (1:2000 in the nonfat milk). Two hours later, the immunoreactive protein was detected using ECL and Gel Imager (BioRad, Hercules, CA, USA). The bands were analyzed with ImageLab software (version 5.2.1, BioRad, Hercules, CA, USA). Western blots were performed 3 times.

### 2.10. Real-Time PCR Assay

The total RNA was isolated from the RVECs using a TRIzol reagent (Invitrogen, Carlsbad, CA, USA). The RNA was reverse-transcribed to complementary DNA (cDNA) by a superscript Reverse Transcription kit (Roche, Basel, Switzerland). Oligo7 software (version 7.56, Molecular Biology Insights, Vondelpark Colorado Springs, CO, USA) was used to select specific primer sequences. qRT-PCR samples were prepared in a 10 µL of mixture, which consisted of Fast Start Universal SYBR Green Master, cDNA and primers. β-actin was used as an internal control. The relative expression levels of target genes were calculated using 2^−ΔΔCT^ method. All the primers were detailed in Table 1 and the thermocycling conditions were performed in Table 2.

### 2.11. Statistical Analysis

SPSS 20.0 software (SPSS Inc., Chicago, IL, USA) was used to perform statistical analysis. All the data were shown as means ± SD. The differences of groups were examined by One-way analysis of variance (ANOVA). If the variance was homogeneous, the differences among groups were analyzed by least significant difference (LSD). Otherwise, the results were analyzed by Tamhan’s T2. The differences were considered to be significant when the *p* value is less than or equal to 0.05.

## 3. Results

### 3.1. The Cytotoxicity of Folic Acid

**Figure 1:**
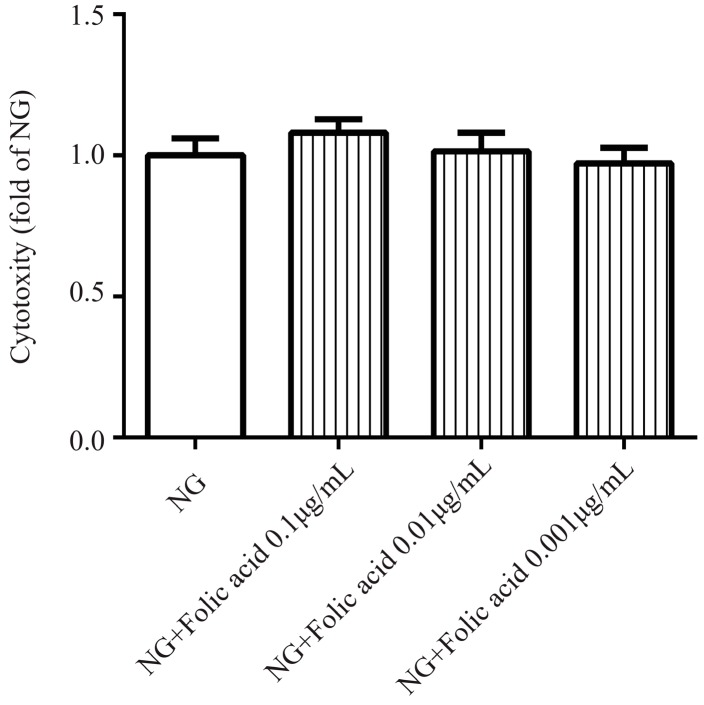
Folic acid had no toxic effect on RVECs. The cells (4000 per/well) were treated with NG or various concentrations of folic acid for 72 h. The abbreviation NG represents normal glucose, i.e., the cells were cultured in a medium with a glucose concentration at 5.5 mM. The effect of folic acid (0.1–0.001 μg/mL) on RVECs cytotoxicity was assessed by CCK-8 assay. The data was presented as means ± SD (*n* = 6).

The toxic concentration of folic acid was confirmed using the CCK-8 assay. Folic acid did not show any toxicity in the RVECs when the dose reached the indicated concentration at 72 h (Figure 1). Its non-toxic concentration varied from 0.1 μg/mL to 0.001 μg/mL.

### 3.2. The Effects of Folic Acid on the Viability of High Glucose (HG)-Treated RVECs

**Figure 2:**
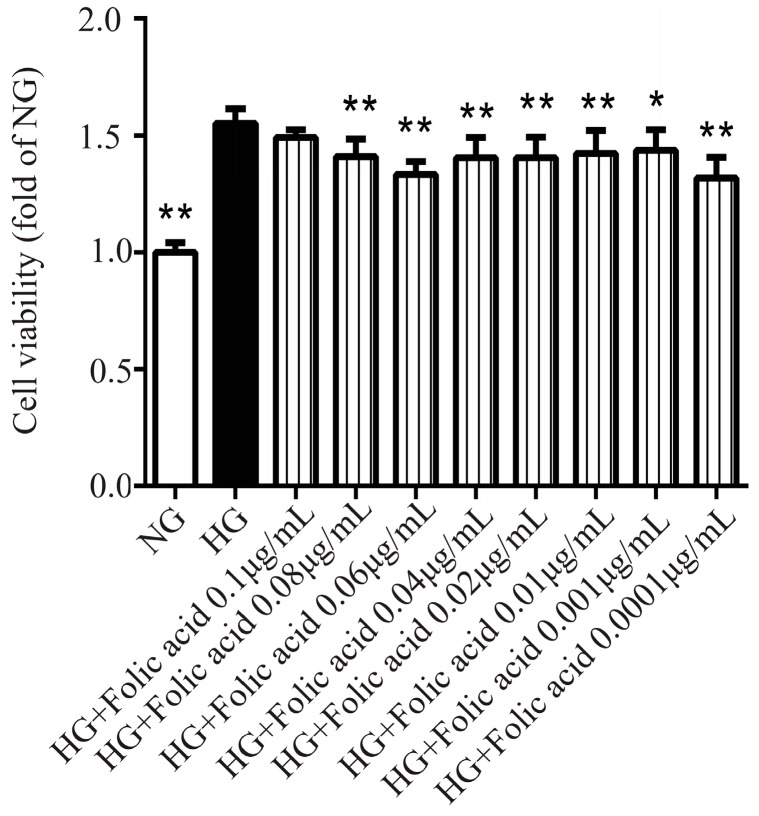
Folic acid inhibited the cell viability of RVECs. The cells (4000 per/well) were treated with HG or various concentrations of folic acid for 72 h. The abbreviation HG represents high glucose, i.e., the cells were cultured in a medium with a glucose concentration at 25 mM. The effect of folic acid (0.1–0.0001 μg/mL) on RVECs viability was assessed by CCK-8 assay. The data was presented as means ± SD (*n* = 5–6). * *p* < 0.05, ** *p* < 0.01, compared with HG-treated cells.

Compared with the normal glucose (NG), the high glucose increased the viability of RVECs (Figure 2), which was consistent with the literature [15,16]. Folic acid (Figure 2) significantly suppressed the viability of the RVECs. Moreover, folic acid showed a promising effect. It reduced the cell viability by 42%, and its effective doses started from 0.0001 to 0.08 μg/mL.

### 3.3. The Effects of Folic Acid on the Migration of HG-Treated RVECs

**Figure 3:**
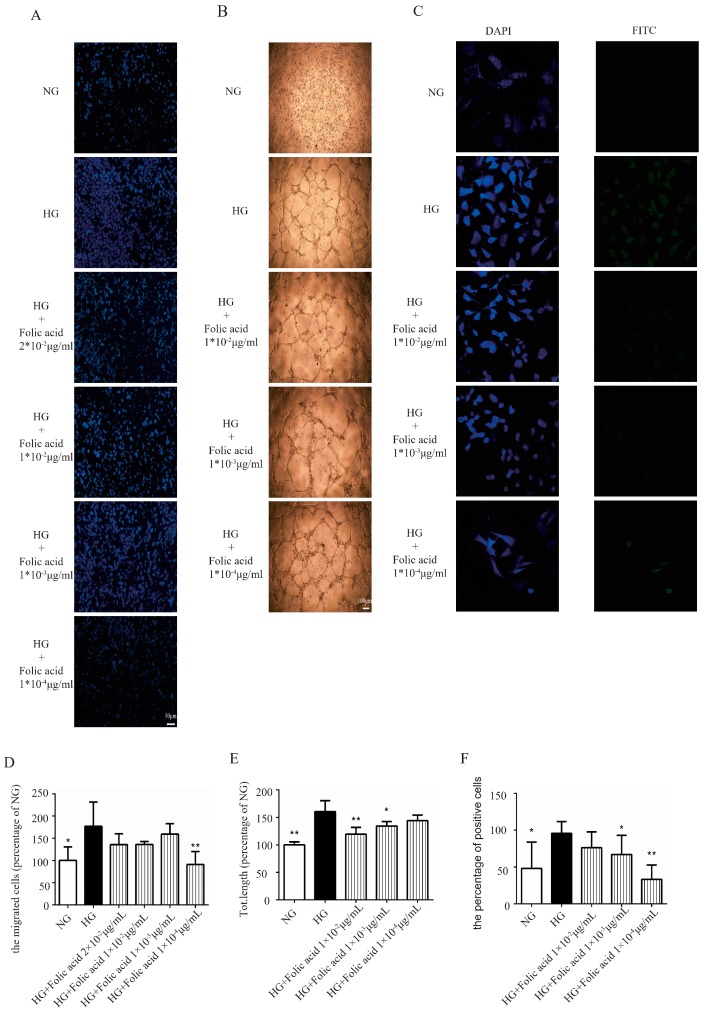
Folic acid inhibited the migration capability, total tube length and proliferation of the RVECs. The effect of folic acid (0.02–0.0001 μg/mL; **A**,**D**) on the migration of RVECs was assessed by the Transwell assay. Scale bar, 50 μm. The effect of folic acid (0.01–0.0001 μg/mL; **B**,**E**) on the tube formation of RVECs was assessed by the tube formation assay. Scale bar, 100 μm. The effect of folic acid (0.01–0.0001 μg/mL; **C**,**F**) on the proliferation of RVECs was assessed by BrdU assay. The data was presented as means ± SD (*n* = 3–12). * *p* < 0.05, ** *p* < 0.01, compared with HG-treated cells.

As shown in Figure 3, HG increased the migration of the RVECs (Figure 3) and the migration was markedly suppressed by folic acid with an inhibition rate of 111.91% (Figure 3A,D). This indicated that folic acid had the advantage of controlling the migration capability.

### 3.4. The Effects of Folic Acid on the Tube Formation of HG-Treated RVECs

Matrigel assay is usually used for evaluating the angiogenesis in vitro. Compared with NG, HG significantly increased the total tube length (Figure 3), while folic acid decreased the total tube length compared with HG-treated group (Figure 3B,E). The highest inhibition rate of folic acid was 68.06%.

### 3.5. The Effects of Folic Acid on the Proliferation of HG-Treated RVECs

Furthermore, we tested the effect of folic acid on the cell proliferation using a BrdU assay. As expected, folic acid attenuated the proliferation of RVECs significantly (Figure 3C,F).

### 3.6. The Interaction of Folic Aicd with TEAD1, YAP1, DLL1 and Notch2

The Hippo and Notch signaling pathways are involved in angiogenesis, so we observed whether folic acid could regulate the Hippo and Notch signaling pathways. The Hippo and Notch signaling pathways are composed of various types of proteins, so we used molecular docking to speed up the process. The results of molecular docking showed that Notch2, DLL1, TEAD1 and YAP1 exhibited the highest binding scores, so they were selected for the verification (Table 3).

### 3.7. The Effects of Folic Acid on the mRNA Expression of TEAD1 and DLL1

Since folic acid exhibited the desired pharmacological activity according to the cell viability, migration, tube formation and proliferation, its underlying mechanism was further investigated.

**Figure 4:**
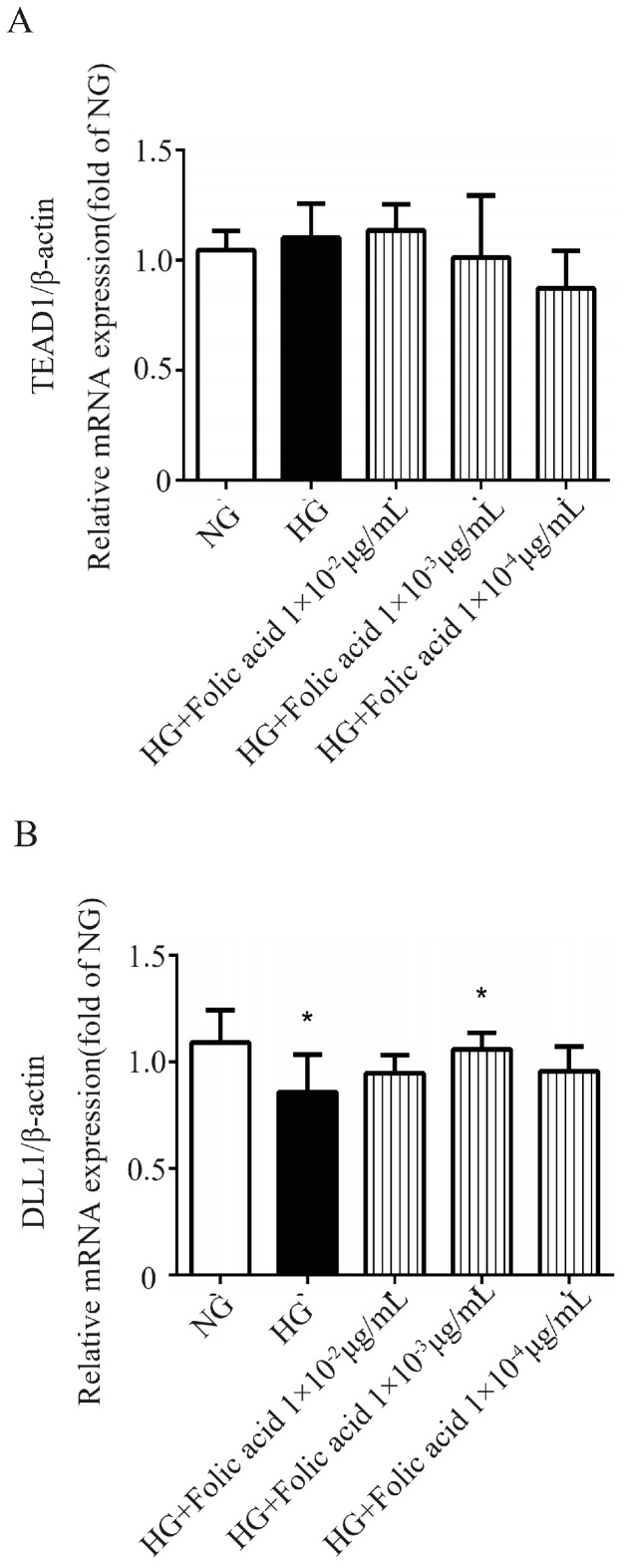
Folic acid decreased the TEAD1 mRNA and increased the DLL1 mRNA level in the RVECs. The cells were treated with 1 × 10^−2^ μg/mL, 1 × 10^−3^ μg/mL, 1 × 10^−4^ μg/mL of folic acid for 72 h. The mRNA levels of TEAD1 (**A**) and DLL1 (**B**) were assessed by Real-Time PCR. The data was presented as means ± SD (*n* = 4). * *p* < 0.05, compared with HG-treated cells.

According to the molecular docking, the targets of folic acid were TEAD1, YAP1, DLL1 and Notch2. Then the gene levels of the targets in RVECs were examined. As shown in Figure 4, compared with NG, HG promoted the expression of TEAD1 mRNA and inhibited the expression of DLL1 mRNA. After treatment with folic acid, the RVECs presented a lower tendency of TEAD1 mRNA expression (Figure 4A). Besides, folic acid could significantly increase the expression level of DLL1 mRNA (Figure 4B). Nevertheless, there was no difference of YAP1 and Notch2 mRNA expression among the groups (data not shown).

### 3.8. The Effects of Folic Acid on the Protein Expression of TEAD1, YAP1, P-YAP1, DLL1 and Notch2

**Figure 5:**
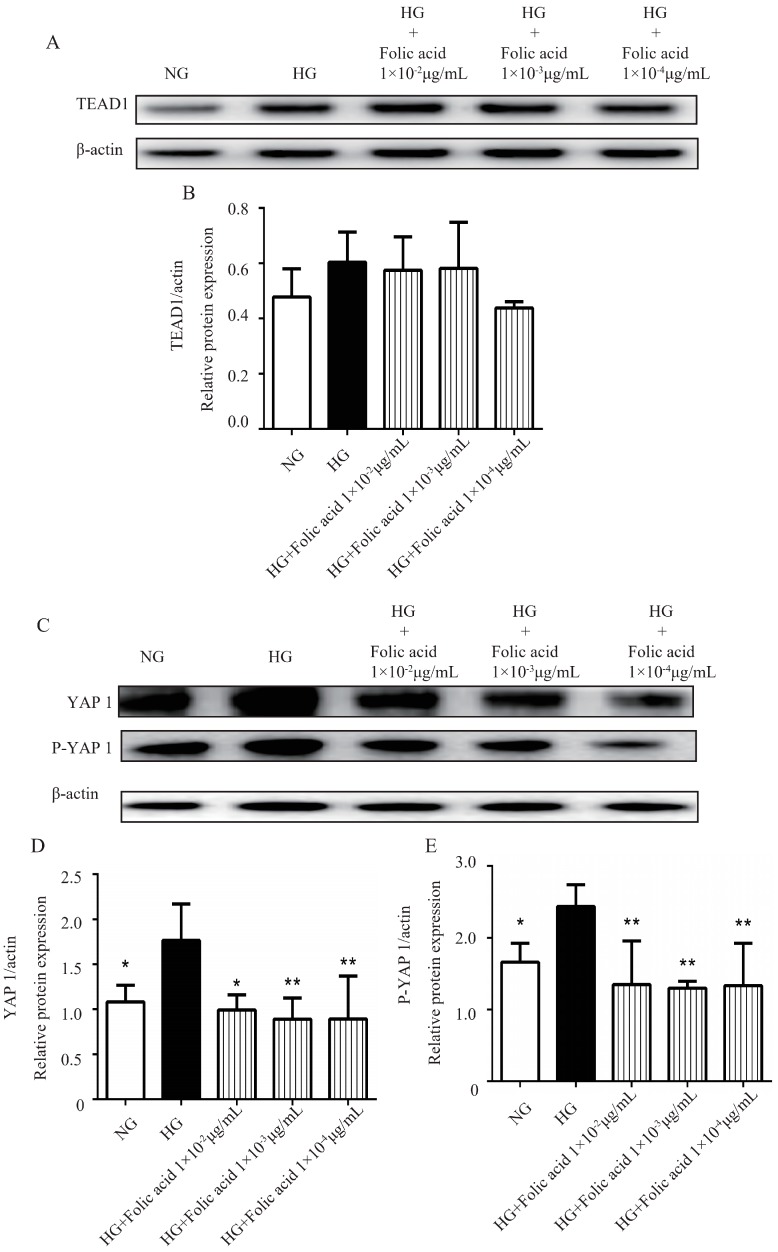
Folic acid attenuated the protein expression of TEAD1 and YAP1 in the RVECs. The cells were incubated with 1 × 10^−2^ μg/mL, 1 × 10^−3^ μg/mL, 1 × 10^−4^ μg/mL of folic acid for 72 h. The protein expression levels of TEAD1 (**A**,**B**), YAP1 (**C**,**D**) and P-YAP1 (**C**,**E**) were assessed by western blot. The data was presented as means ± SD (*n* = 3). * *p* < 0.05, ** *p* < 0.01, compared with HG-treated cells.

**Figure 6:**
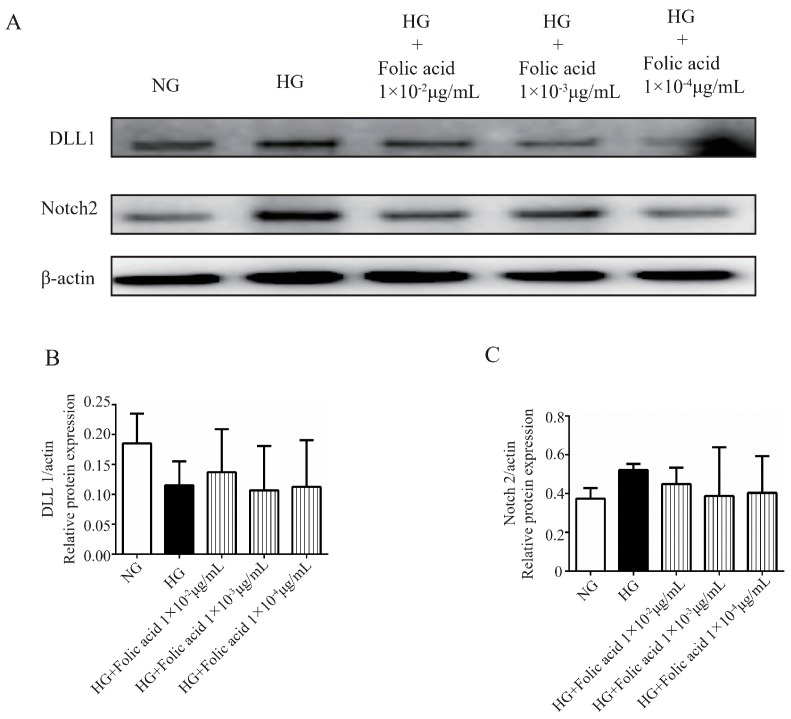
Folic acid promoted the protein expression of DLL1 and attenuated the protein expression of Notch2 in the RVECs. The cells were incubated with 1 × 10^−2^ μg/mL, 1 × 10^−3^ μg/mL, 1 × 10^−4^ μg/mL of folic acid for 72 h. The protein expression levels of DLL1 (**A**,**B**) and Notch2 (**A**,**C**) were assessed by western blot. The data was presented as means ± SD (*n* = 3).

To determine whether folic acid affected the protein expression of TEAD1, YAP1, P-YAP1, DLL1 and Notch2, western blot assays were performed. When the RVECs were grown in HG medium, the protein expression of YAP1 (Figure 5C,D) and P-YAP1 were increased (Figure 5C,E). After the treatment with the different doses of folic acid for 72 h, the protein expression of YAP1 (Figure 5C,D) and P-YAP1 (Figure 5C,E) were significantly decreased. Moreover, the RVECs grown in HG produced a higher tendency of TEAD1 (Figure 5A,B) and Notch2 (Figure 6A,C) protein levels compared with the cells grown in NG medium. Folic acid could downregulate the protein expression of TEAD1 (Figure 5A,B) and Notch2 (Figure 6A,C). The RVECs grown in HG medium produced a lower tendency of DLL1 protein level compared with the cells grown in NG medium. However, folic acid didn’t affect the protein expression of DLL1 (Figure 6A,B). Combining all the results, folic acid could inhibit the protein expression of the Hippo signaling pathway.

## 4. Discussion

The aim of our study was to find out whether folic acid has a protective effect on RVECs against high glucose and explore its molecular mechanism of action. The key results indicated two points. Firstly, folic acid could inhibit the cell viability, cell migration and total tube length in varying degrees. Secondly, folic acid exhibited an important influence on the expression level of proteins in the Hippo signaling pathway. In general, folic acid could probably control the activity of RVECs by regulating the proteins in the Hippo signaling pathway.

There is also some other evidence which shows folic acid has an anti-proliferation effect. It has been reported that folic acid can inhibit the proliferation of vascular smooth muscle cells (VSMCs) induced by platelet derived growth factor (PDGF-BB) [17]. In addition, folic acid depresses the proliferation of human umbilical venous endothelial cells (HUVECs) through activating the cSrc/ERK 2/NF-κB/p53 pathway [18]. It also attenuated the migration and the tube formation of HUVECs by inhibiting RHoA activity [19]. Furthermore, our data suggested that folic acid could suppress the proliferation of RVECs by 33.33%. Therefore, these findings indicate that folic acid has an inhibition effect on the proliferation and migration of the endothelial cells or VSMC under different pathological stimulation.

However, some findings are contrary to our results. For example, Petersen et al. found that folic acid increased the growth rate of LNCaP and PC-3 cells [20]. It was also reported that folic acid stimulated the proliferation of neural stem cells (NSC) at a dose-dependent manner [21]. In addition, high concentration of folic acid promoted the HT-29 cell proliferation [22]. This opposite data may be due to the differences of cell types. Based on all these findings, it is inferred that folic acid is likely to have dual influence on the different cells. And our research serves as a complementary to the overall knowledge of folic acid.

After evaluating the activity of folic acid, the proteins in the Hippo signaling pathway were selected to verify the molecular docking and to clarify the pharmacological mechanism. According to the results of docking, YAP1 and TEAD1 were considered to be the targets of folic acid. Both YAP1 and TEAD1 are members of the Hippo signaling pathway, which are key regulators of organ size and regeneration. Many studies have shown that YAP1 is closely associated with the proliferation, migration and tube formation in different types of cells. Downregulation of YAP could inhibit the growth of Eca-109 cells [23], the endothelial cells [24] and the pancreatic cancer cells [25]. Moreover, overexpression of YAP could facilitate the migration of the bladder cancer cells, KGN granulosa cell tumor cells and SH-SY5Y cells [26,27,28]. In addition endothelial YAP plays an important role in stimulating sprouting angiogenesis [29].

As a major transcription factor partner of YAP, TEADs family brought YAP to certain gene promoters [30,31,32,33,34]. The cell growth will be inhibited when the YAP-TEAD complex is suppressed [35,36]. It has been proved that overexpression of TEAD1 increased the cellular proliferation in colorectal cancer (CRC) [37]. On the contrary, the cell proliferation is reduced in mice embryo after the removal of TEAD1/2 [38].

In consideration of the essential role of YAP and TEAD, the effect of folic acid on these two proteins was observed. The virtual experiment reported that folic acid docked with YAP1 and TEAD1 with high affinity. Moreover, the cells treated with folic acid significantly decreased the expression of YAP1 and P-YAP1 compared with HG groups. In addition, folic acid also diminished TEAD1 protein expression level. In conclusion, folic acid could modulate pathological changes of retinal vascular endothelial cells by regulating YAP1 and TEAD1.

Taken together, folic acid probably can protect the retinal vascular endothelial cells from HG-induced injury by regulating the TEAD1 and YAP1 protein. Our findings not only help to figure out the material mechanism of folic acid, but also provide a new thought about folic acid for future research.

## 5. Conclusions

Folic acid can protect the retinal vascular endothelial cells from HG-induced injury by regulating the proteins from Hippo signaling pathway.

## Figures and Tables

**Table 1:** Primers for quantitative polymerase chain reaction.

Sequences	Sequences, 5’–3’
TEAD1	Forward: ACCTCTTGGCAGTACAGTATTC
	Reverse: CACTTTAAAGCCAACACTTAGAACA
YAP1	Forward: TGAACAAACGTCCAGCAAGATAC
	Reverse: CAGCCCCCAAAATGAACAGTAC
DLL1	Forward: TGAACGACTTCTCCTGCACC
	Reverse: GATGCTTCTCCACTGCTGACG
Notch2	Forward: CTGGTGCCTATTGTGACGTG
	Reverse: TTCAACAAGCACACCTCTCCT
β-actin	Forward: AGCCATGTACGTAGCCATCC
	Reverse: TCTCAGCTGTGGTGGTGAAG

**Table 2:** Thermocycling program for quantitative polymerase chain reaction.

Step	Temperature, °C	Duration
1	95	10 min
2	95	15 sec
3	60	60 sec
Steps 2–3	-	40 cycles

**Table 3:** The partial results of the molecular docking.

Component	Signaling Pathway	Target Protein	Docking Scores
folic acid	Hippo	TEAD1	−8.84
		YAP1	−6.08
	Notch	DLL1	−6.25
		Notch2	−5.77

## Data Availability

The raw data supporting the conclusions of this manuscript will be made available by the authors, without undue reservation, to any qualified researcher.

## Abbreviations

DR	Diabetic retinopathy
PDR	Proliferative diabetic retinopathy
NPDR	Nonproliferative diabetic retinopathy
DWR	Diabetic without retinopathy
RVEC	Retinal vascular endothelial cell
OD	Optical density
DAPI	4′,6-Diamidino-2-phenylindole
RT	Room temperature
PDB	Protein Data Bank
SDS	Sodium dodecyl sulfate
DDT	Dichlorodiphenyltrichloroethane
cDNA	Complementary DNA
ANOVA	One-way analysis of variance
LSD	Least significant difference
VSMC	Vascular smooth muscle cell
PDGF-BB	Platelet derived growth factor
HUVEC	Human umbilical venous endothelial cell
NSC	Neural stem cell
CRC	Colorectal cancer
NG	Normal glucose
HG	High glucose
